# A pre- and post-intervention study testing the effect of exposure to languageless animated images communicating COVID-19 preventive behaviours on behavioural intentions and beliefs of Guatemalan adults

**DOI:** 10.7189/jogh.12.05018

**Published:** 2022-07-22

**Authors:** Nicola O’Brien, Santosh Vijaykumar, Michael Craig, Ellie Land, Sigrid M Aquilar Jocol, Xiomara G Bedoya Mendoza, Rony de la Cruz Estrada, Edwin A Najera Gonzalez, Luisa F Nicolau Ozaeta

**Affiliations:** 1 Department of Psychology, Northumbria University, Newcastle upon Tyne, UK; 2 Department of Arts, Northumbria University, Newcastle upon Tyne, UK; 3 The Human Rights Office of the Archbishop of Guatemala (Oficina de Derechos Humanos del Arzobispado de Guatemala, ODHAG), Guatemala

## Abstract

**Background:**

Effective health communication to encourage participation in COVID-19 preventive behaviours is crucial in helping mitigate viral spread. Intentions and beliefs are known determinants of adherence to these behaviours, therefore, health communication interventions based on these constructs may be effective. Visual languageless messages can be particularly useful in multilingual countries, where text-based communications can limit message exposure. This pre- and post-intervention study sought to identify the effect of exposure to languageless animated messages, presented in the Graphic Interchange Format (GIF), communicating COVID-19 preventive behaviours (physical distancing, handwashing, and mask-wearing) on behavioural intentions and beliefs.

**Methods:**

Between February and March 2021, a nationally representative sample of 308 Guatemalan adults completed this online survey experiment. Self-reported performance of preventive behaviours, understanding of COVID-19 transmission risk, as well as intentions, self-efficacy, and outcome expectancy beliefs about preventive behaviours were assessed at baseline. Participants were then exposed to a random combination of three of four possible GIFs in random presentation order. Following exposure to each GIF, intentions, self-efficacy, and outcome expectancy beliefs were reassessed.

**Results:**

In terms of main effects, GIF exposure was significantly associated with improved intentions, self-efficacy, and outcome expectancy beliefs in relation to physical distancing; intentions and outcome expectancy beliefs in relation to handwashing; and intentions and self-efficacy in relation to mask-wearing. These associations were not dependent on the combination of the three of four possible GIFs presented. Pairwise comparisons revealed that observed improvements in scores were most pronounced from baseline to the first GIF exposure and reduced thereafter.

**Conclusions:**

Exposure to languageless GIFs communicating COVID-19 preventive behaviours is associated with improvements in key social-cognitive determinants of those behaviours. Dosage of GIF exposure and durability of effects are issues that warrant further attention so we can better understand the conditions and point at which benefits are maximised. Moreover, the effect on behavioural adherence is yet to be determined. GIFs provide a valuable means to widely disseminate health messages via social media during public health crises, such as COVID-19. When these messages are languageless, the potential reach of dissemination can be maximised.

Effective public health communications are critical in preventing the spread of COVID-19. Globally, government guidance and legislation have advocated and coerced evidence-based transmission preventive behaviours including physical distancing, good hygiene practices such as handwashing, and mask-wearing. Encouraging individual adherence to these behaviours is challenging, requiring input and evidence from behavioural science [1,2].

Increasing knowledge through information provision is generally considered necessary, but insufficient for health behaviour change [3,4]. Research on the individual determinants of transmission-preventive behaviours provides evidence of other potentially modifiable targets for behaviour change interventions to help during the COVID-19 pandemic. Intention, self-efficacy (ie, confidence in performing the behaviour) and outcome expectancy or behavioural efficacy (ie, anticipated consequences of the behaviour) have been shown to predict preventive behaviours of physical distancing (ie, maintaining 1-2 m of distance from people in other households), handwashing and mask-wearing [5-12].

Social Cognitive Theory (SCT) [13] is suitable for understanding how such beliefs impact adherence to COVID-19 preventive behaviours and provides a theoretical basis for behaviour change interventions designed to modify them. Self-efficacy, a key construct of SCT, is an important predictor of behaviour; the more confident an individual feels about engaging in a preventive behaviour, the more likely they will engage in it. Outcome expectancy, another key construct within SCT, is the belief that the behaviour will yield a particular result. SCT proposes that providing opportunities for individuals to instil expectations and self-efficacy by obtaining mastery and vicarious (modelling) experiences can produce behaviour change [14]. Self-efficacy can predict behavioural intentions to engage in COVID-19 preventive behaviours [15-16], and intentions often, but not always, predict behaviour performance [17].

Evidence suggests that information is better retained when health communications include visuals rather than text alone [18]. Unlike text-based health communications, visual communications do not rely on language but use images, animations, and videos to tell the message narrative. One such type of animated communication is the Graphics Interchange Format (GIF), a digital file format frequently used within digital communication with endless looping repetition and no sound. GIFs are easily shared within internet-based communication due to their small file size [19]. The important global role of languageless, animated messages promoting COVID-19 preventive behaviours has been recognised [20] and preliminary evidence has demonstrated its positive impact on behaviour change [21]. In countries with multiple official languages, languageless animated communications may provide a solution to effectively disseminate messages to the entire population.

Guatemala is one such country, with 25 official languages spoken (24 indigenous and Spanish). It is the most populous country in Central America with significant socioeconomic inequalities, a weak underfunded public health system, and some of the worst health issues globally [22,23]. As of January 7, 2022, Guatemala is one of the Central American countries most affected by COVID-19 with 631 730 confirmed cases, 16 114 deaths and 28.5% of the population fully vaccinated [24].

The present study aimed to identify the effect of exposure to languageless messages, in the form of original animated GIF images, communicating COVID-19 preventive behaviours on intentions, self-efficacy, and outcome expectancy beliefs in relation to physical distancing, handwashing and mask-wearing behaviours. We hypothesised that GIF exposure would be associated with improvements in intentions and beliefs about preventive behaviours.

## METHODS

### Study design

This study used a pre- and post-intervention repeated measures design.

### Sample

Data were collected via an online survey in Spanish. Adults (18+ years) who lived in Guatemala and spoke Spanish, were eligible to participate and were recruited through Qualtrics^XM^ panel of respondents. Demographic quotas based on the 2018 census of Guatemala (ie, males = 58%; 18-24 years = 35%, 25-34 years = 41%, 35-44 years = 16%, 45-54 years = 6%, 55+ years = 2%) were used to provide a representative sample. Data collection occurred between February 18 and March 8, 2021, using the QualtricsXM survey platform (Version February 2021; Qualtrics, 2005; Provo, Utah, USA). At that time, Guatemala’s COVID-19 recommendations included keeping 1.5 m away from others, wearing a mask in public places and frequent handwashing with soap and water.

### Materials

**Figure 1:**
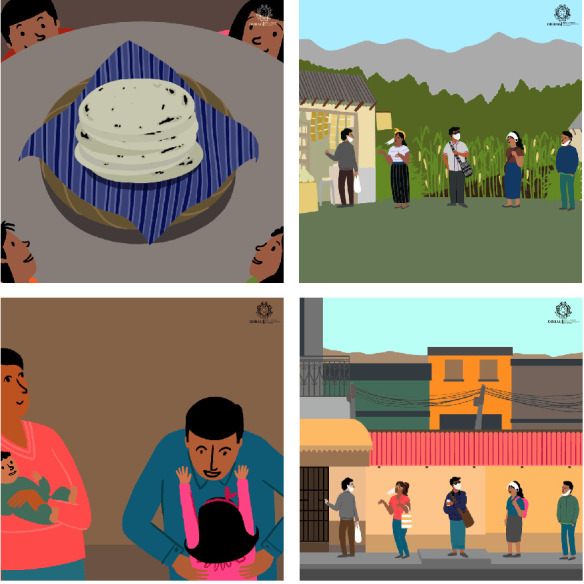
The GIFs focussing on families or youths, centred either within a rural or urban context. GIF 1 Rural family; GIF 2 Rural youth; GIF 3 Urban family; GIF 4 Urban youth. Available at: https://www.youtube.com/channel/UCbeGGPYy1PDdb3xLHC_jTSw

Four languageless GIFs were developed iteratively between June and December 2020. GIFs are usually created by taking a small section from existing media; however, the GIFs in this study were original artworks designed by the research team. Evidence from a review of existing COVID-19 visual health communications, qualitative interviews with professionals, and behaviour change and health communication literature was integrated with stakeholder involvement through a series of co-design workshops [25]. The GIFs were based around SCT, expected to increase intentions, self-efficacy, and outcome expectancies about physical distancing (hereafter referred to as “distancing”), handwashing, and mask-wearing. The four GIF designs and narratives were developed to be culturally relevant and sensitive to the Guatemalan population; they recognise high collectivism, focus on families or youths, and are centred within a rural (total = 2) or urban (total = 2) context (see **Figure 1**). Each participant was presented with three of the four GIFs. GIF selection was randomised automatically: Combination 1 (GIFs A, B, C), n = 80/308 (26%); Combination 2 (GIFs A, B, D), n = 75/308 (24%); Combination 3 (GIFs A, C, D), n = 82/308 (26%); Combination 4 (GIFs B, C, D), n = 71/308 (23%).

### Measures

Intentions, self-efficacy, and outcome expectancy beliefs about each of the COVID-19 preventive behaviours were probed using questions based upon previously used measures [8]. Intention was measured on a 5-point Likert scale (always, most times, sometimes, rarely, never; scale range 1-5), self-efficacy was measured on a 4-point Likert scale (very confident, fairly confident, not very confident, not at all confident; scale range = 1-4) and outcome expectancy was measured on a 4-point scale (strongly agree, tend to agree, tend to disagree, strongly disagree; scale range = 1-5). “Don’t know” and “Prefer not to say” were alternative available responses. No items were reverse coded; a lower score reflected a stronger intention and belief.

For distancing and handwashing behaviours, the following single items were used:

1. Intention – “How often do you intend to stay 1.5 metres away from other people, except those who live in your household, all or most of the time?” and “How often do you intend to wash your hands as soon as you get home?”;
2. Self-efficacy – “How confident are you that you can stay 1.5 metres away from other people, except those who live in your household, all or most of the time?” and “How confident are you that you can wash your hands as soon as you get home?”;
3. Outcome expectancy – “How much do you agree with the statement “I will reduce my chances of getting COVID-19” in relation to washing your hands as soon as you get home?” And “In relation to keeping a 1.5-metre distance from people outside of your household?”.

Single items for intentions and beliefs have been found to be psychometrically sound and favourable to multiple item measures reducing monotony and burden on participants [26,27].

For mask-wearing behaviour, intention, self-efficacy. and outcome expectancy were checked using the same question format but applied to four common behavioural settings:

1. Outside of your home;
2. On public transport;
3. In the street with friends or family;
4. When other people around you are NOT wearing a face mask correctly.

Cronbach’s reliability analyses confirmed acceptable reliability between mask-wearing sub-scores in each testing phase for each type of belief: intention α>0.697, self-efficacy α>0.771, and outcome expectancy α>0.801, in all cases. To this end, for each phase, sub-scores were averaged into single mean scores for analyses. Attitudes to the behaviours were also elicited but data are not reported here.

### Procedure

The survey was drafted in English and then translated into Spanish and back-translated by research team members fluent in English and Spanish, including a language expert. The Guatemala-based team provided feedback on the clarity of questions and accuracy concerning government recommendations at that time. The survey was conducted using Qualtrics^XM^ and piloted in 10% of the target sample to ensure accuracy of data collection before continuing recruitment.

Participants first rated their engagement in preventive behaviours (distancing, handwashing, and mask-wearing) during the week preceding the study. Understanding of COVID-19 threat was probed along with knowledge of COVID-19 transmission risk from different situations (eg, not washing hands). Intentions, self-efficacy, and outcome expectancies in relation to distancing, handwashing, and mask-wearing, in that order, were assessed.

Participants were then presented with three GIFs sequentially. Using the Qualtrics^XM^ platform, the presented GIFs were randomly selected from a set of four (**Figure 1**) and their delivery order was randomised. GIF viewing was self-paced, and no time limit was placed on subsequent completion of the intention and belief measures. Following each GIF, participants completed the same intention and belief measures as they did at baseline. Post-GIF measurements were collected on three occasions. Further questions explored which GIF participants liked the most, liked the least, and narrative or emotive responses; these data are not reported here.

### Analyses

A priori power calculation using G*Power (Version 3.1.9.7; 2020) yielded a sample size of 148 to detect within-subject, between-subject, and interaction effects, a medium effect size (f = 0.25), 0.05 probability of error and 0.90 power. Other analyses were performed using IBM SPSS (Version 26.0; IBM Corp, 2019; Armonk, NY). Response options “Don’t know” and “Prefer not to say” were treated as missing values and removed. There were 351 missing values, which accounted for 1.19% of all data points. Repeated measures (RM) ANOVAs with within-subject factor GIF exposure (baseline, GIF1, GIF2, GIF3) were performed to examine possible changes in responses to intention, self-efficacy, and outcome expectancy measures. As each participant was presented with three randomly selected GIFs of a possible four, GIF combination was included in all RMANOVAs as a between-subject factor. In all cases, reporting of RMANOVA outcomes include *F* (variation between sample means), *df* (degrees of freedom), *P* (probability), and ηp^2^ (partial eta squared effect size) values, eg, (*F* (3903) = 53.000, *P* < 0.001, ηp^2^ = 0.150). Planned pairwise comparisons examined possible differences in scores between testing phases, eg, baseline vs post-GIF1. Reporting of *t* test outcomes included a *t* (difference between means), *df* and *P-*values, eg, (*t* (303) = 0.232, *P* = 0.817). A Bonferroni-corrected alpha level of *P* = 0.008 (*P* = 0.05/6 pairwise tests) was applied to account for multiple comparisons. These corrections were applied to within-family pairwise comparisons, eg, for the “intention” measure of distancing or “outcome expectancy” measure of mask-wearing. Significant outcomes that survived Bonferroni correction are denoted in the results section through an asterisk, eg, “*P* = 0.003*”.

### Ethical approval

This study was approved by Northumbria University Research Ethics Committee (Ref: 26593). All procedures performed in studies involving human participants were in accordance with the ethical standards of the institutional and/or national research committee and with the 1964 Helsinki declaration and its later amendments or comparable ethical standards.

## RESULTS

### Sample characteristics

A total of 308 adults (mean age = 29.69 years, SD = 9.11; range = 18-62; 58.4% male) participated in this study. Most participants identified as Latino (82.5%) with others identifying as Maya (10.7%), Garifuna (2.3%), and Xinca (1.0%). Spanish was the dominant mother language reported by 93.5% of participants. Participants reported living in their household with a mean of 3.16 adults aged ≥18 years (SD = 1.92; range = 0-15) and 1.27 children aged <18 years (SD = 1.38; range = 0-13). 138 participants (44.8%) reported being in full or part-time employment, 17.5% were self-employed, 13.6% were studying at school, college or university, and 23.1% were not employed (see **Table 1** for sample data).

**Table 1:** Sample characteristics

Variable	Categories	n (%)	Variable	Categories	n (%)
Age	18-24 y	107 (34.7%)	Employment status	Full- or part-time	138 (44.8%)
	25-34 y	127 (41.2%)		Self-employed	54 (17.5%)
	35-44 y	50 (16.2%)		Unemployed	56 (23.1%)
	45-54 y	22 (7.1%)		Studying	42 (13.6%)
	55+ years	4 (1.3%)		Looking after the home	10 (3.2%)
				Retired	1 (0.3%)
Gender	Male	180 (58.4%)		Not working due to disability or illness	4 (1.3%)
	Female	123 (39.9%)		Prefer not to say	3 (1.0%)
	Other	1 (0.4%)			
	Prefer not to say	4 (1.3%)	Adults in household	0	24 (7.8%)
				1	19 (6.2%
Self-identified ethnicity	Latino	254 (82.5%)		2	75 (24.4%)
	Maya	33 (10.7%)		3	61 (19.8%)
	Garifuna,	7 (2.3%)		4	65 (21.1%)
	Xinca	3 (1.0%)		5	33 (10.7%)
	Prefer not to say	2 (0.6%)		6	11 (3.6%)
				7	4 (1.3%)
Mother language	Spanish	288 (93.5%)		8	5 (1.6%)
	Achi’	1 (0.3%)		9	1 (0.3%)
	K’iche’	3 (1.0%)		10+	1 (0.3%)
	Mam	2 (0.6%)			
	Poqomchi’	1 (0.3%)	Children in household	0	90 (29.2%)
	Q’eqchi’	1 (0.3%)		1	98 (31.8%)
	Tz’utujil	1 (0.3%)		2	65 (21.1%)
	Garifuna	3 (1.0%)		3	24 (7.8%)
	Kaqchikel	6 (1.9%)		4	8 (2.6%)
	Ixil	1 (0.3%)		5+	4 (1.2%)

### Pre-study self-reported performance of preventive behaviours

In the week preceding the study, 220 participants (71.4%) reported leaving their homes at least once. Among those, 45.9% reported *always* performing distancing, 85.0% handwashing and 90.9% mask-wearing (see **Table 2**).

**Table 2:** Self-reported performance of preventive behaviours in the week preceding the study in the subset (n = 220) of participants who reported having left their home at least once

	How often did you engage in these behaviours in the past week?						
**Behaviour**	**Always**	**Most times**	**Sometimes**	**Rarely**	**Never**	**Prefer not to say**	**Total n of subset**
Stayed 1.5m away from people outside of home	101 (45.9%)	90 (40.9%)	19 (8.6%)	7 (3.2%)	2 (0.9%)	1 (0.5%)	220
Stayed 1.5m away from all people	42 (19.1%)	46 (20.9%)	43 (19.5%)	52 (23.6%)	28 (12.7%)	9 (4.1%)	220
Wore a mask correctly when outside of home	200 (90.9%)	14 (6.4%)	4 (1.8%)	1 (0.5%)	0 (0.0%)	1 (0.5%)	220
Washed your hands as soon as you got home	187 (85.0%)	19 (8.6%)	11 (5.0%)	2 (0.9%)	0 (0.0%)	1 (0.5%)	220

### Pre-study understanding of COVID-19 behavioural transmission risks

In relation to understanding the risks of COVID-19 transmission, participants felt that *too much contact with others* (76.9%), *not washing hands enough* (67.5%) and *not wearing a mask correctly* (80.8%) increased their risk to a great extent (see **Table 3**).

**Table 3:** Pre-study understanding of risk of COVID-19 transmission by behaviours

	Would the behaviour increase your risk of contracting COVID-19?						
**Risk behaviour**	**To a great extent**	**To some extent**	**Hardly at all**	**Not at all**	**Don't know**	**Prefer not to say**	**Total n**
Too much contact with others	237	58	7	3	3	0	308
Not washing hands enough	208	82	6	9	2	1	308
Not wearing a mask correctly	249	39	6	12	2	0	308
A member of family brought it home	113	56	13	116	8	2	308
Others didn't keep distance when out of the home	132	126	14	22	11	3	308

### Effect of GIF exposure on intentions and beliefs in relation to preventive behaviours

**Table 4** provides an overview of all RMANOVA main and interaction effects for the distancing, handwashing, and mask-wearing measures.

**Table 4:** Overview of RMANOVA main and interaction effects for the three measures, distancing, handwashing, and mask-wearing†

	GIF exposure‡	GIF combination	GIF exposure × Gif combination
**Distancing**			
Self-efficacy	F (3903) = 53.000, *P* < 0.001*	F (3301) = 0.841, *P* = 0.472	F (9903) = 1.628, *P* = 0.122
Intention	F (3897) = 39.752, *P* < 0.001*†	F (3299) = 0.146, *P* = 0.932	F (9897) = 2.307, *P* = 0.026†
Outcome expectancy	F (3900) = 16.907, *P* < 0.001*†	F (3300) = 0.384, *P* = 0.764	F (9300) = 0.859, *P* = 0.542
**Handwashing**			
Self-efficacy	F (3909) = 3.448, *P* = 0.025†	F (3303) = 0.376, *P* = 0.771	F (9909) = 0.714, *P* = 0.664
Intention	F (3903) = 3.113, *P* = 0.026†	F (3301) = 0.725, *P* = 0.538	F (9903) = 0.601, *P* = 0.750
Outcome expectancy	F (3906) = 6.050, *P* = 0.001*†	F (3302) = 1.116, *P* = 0.343	F (9906) = 0.609, *P* = 0.748
**Mask-wearing**			
Self-efficacy	F (3915) = 44.903, *P* < 0.001*†	F (3305) = 0.520, *P* = 0.669	F (9915) = 1.019, *P* = 0.422
Intention	F (3915) = 7.726, *P* < 0.001*†	F (3305) = 1.267, *P* = 0.286	F (9915) = 1.063, *P* = 0.388
Outcome expectancy	F (3900) = 0.769, *P* = 0.508	F (3300) = 0.306, *P* = 0.821	F (9900) = 0.585, *P* = 0.805

*Significant findings that survived a Bonferroni-corrected *P-*value of 0.002 (*P* = 0.05/27 comparisons).

†Significant findings.

#### Distancing

**Figure 2:**
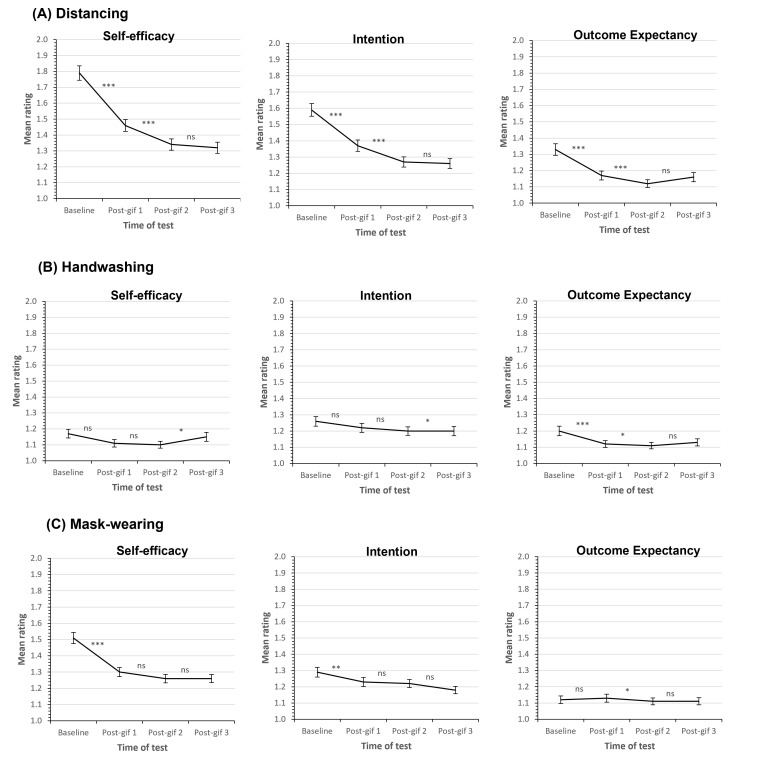
Intentions and beliefs in relation to the behaviours. Line graphs show mean *self-efficacy*, *intention*, and *outcome expectancy* Likert scale ratings in the pre- and post-GIF exposure phases for (**A**) distancing, (**B**) handwashing and (**C**) mask-wearing. In all cases, lower ratings reflect stronger intentions and beliefs. Error bars show the standard error of the mean. Significance values from pairwise comparisons (baseline vs post-GIF1, post-GIF1 vs post-GIF2, and post-GIF2 vs post-GIF3) are shown, where ns = non-significant, * = *P* < 0.05, ** = *P* < 0.01, and *** = *P* < 0.001. Panel A. Distancing; Panel B. Handwashing; Panel C. Mask-wearing.

GIF exposure significantly improved participants’ self-efficacy (*F* (3903) = 53.000, *P* < 0.001, ηp^2^ = 0.150), intentions (*F* (3897) = 39.752, *P* < 0.001, ηp^2^ = 0.117), and outcome expectancy beliefs (*F* (3,900) = 16.907, *P* < 0.001, ηp^2^ = 0.053) (see **Figure 2**, panel A).

#### Handwashing

GIF exposure significantly improved participants’ self-efficacy (*F* (3909) = 3.448, *P* = 0.025, ηp^2^ = 0.011), intentions (*F* (3903) = 3.113, *P* = 0.026, ηp^2^ = 0.010), and outcome expectancy beliefs (*F* (3906) = 6.050, *P* = 0.001, ηp^2^ = 0.020) (see **Figure 2**, panel B).

#### Mask-wearing

GIF exposure significantly improved participants’ self-efficacy (*F* (3915) = 44.903, *P* < 0.001, ηp^2^ = 0.128), and intentions (*F* (3915) = 7.726, *P* < 0.001, ηp^2^ = 0.025). There was no significant effect of GIF exposure on outcome expectancy beliefs (*F* (3900) = 0.769, *P* = 0.508, ηp^2^ = 0.003) (see **Figure 2**, panel C).

### Between subject and interaction effects

GIF combination did not significantly affect ratings of intentions or beliefs in relation to any of the three behaviours (all *P*s >0.471 for distancing; all *P*s >0.342 for handwashing; all *P*s >0.285 for mask-wearing). In addition, no significant interactions between GIF exposure and GIF combination were identified for handwashing (all *P*s >0.663) and mask-wearing (all *P*s >0.387). We did observe a significant interaction for the distancing *intention* behaviour (*P* = 0.026) but this did not survive Bonferroni correction for multiple comparisons (see **Table 4**). Together, these findings indicate that the effects of GIF exposure are unlikely to be dependent on the combination of GIFs presented.

### Post hoc comparisons: exposure-by-exposure changes in ratings

**Figure 2** provides a visual overview of changes in ratings across the measures on an exposure-by-exposure basis, ie, from baseline to post-GIF1, post-GIF2 to post-GIF3 phases of our study procedure.

#### Distancing

Pairwise comparisons revealed that, relative to baseline, participants’ responses were significantly improved in the post-GIF1 (*t* (305) = 7.066, *P* < 0.001*), post-GIF2 (*t* (306) = 9.647, *P* < 0.001*) and post-GIF3 (*t* (305) = 8.981, *P* < 0.001*) phases for self-efficacy (see **Figure 2**, panel A, left); in the post-GIF1 (*t* (303) = 5.570, *P* < 0.001*), post-GIF2 (*t* (304) = 7.903, *P* < 0.001*) and post-GIF3 (*t* (303) = 7.747, *P* < 0.001*) phases for intention (see **Figure 2**, panel A, middle); and in the post-GIF1 (*t* (305) = 5.047, *P* < 0.001*), post-GIF2 (*t* (306) = 5.916, *P* < 0.001*) and post-GIF3 (*t* (304) = 4.266, *P* < 0.001*) phases for outcome expectancy (see **Figure 2**, panel A, right).

Self-efficacy and intention improved significantly from the post-GIF1 to the post-GIF2 phase (self-efficacy: *t* (306) = 3.752, *P* < 0.001*; intention: *t* (306) = 3.838, *P* < 0.001*), but there was no significant change in ratings between the post-GIF2 and post-GIF3 testing points (self-efficacy: *t* (307) = 0.588, *P* = 0.557; intention: *t* (305) = 0.262, *P* = 0.793). Improvement from the post-GIF1 and post-GIF3 phases was significant (self-efficacy: *t* (304) = 3.927, *P* < 0.001*; intention: *t* (304) = 3.927, *P* < 0.001*). In terms of outcome expectancy, there was no significant change in scores from the post-GIF1 to post-GIF2 phases (*t* (306) = 1.814, *P* = 0.071) or post-GIF2 to post-GIF3 phases (*t* (304) = -1.503, *P* = 0.134), nor was there a significant difference between post-GIF1 and post-GIF3 ratings (*t* (303) = 0.232, *P* = 0.817).

#### Handwashing

Pairwise comparisons of intention scores revealed no significant difference between baseline, post-GIF1 (*t* (305) = 1.727, *P* = 0.085) and post-GIF3 (*t* (305) = 01.897, *P* = 0.059) scores (see **Figure 2**, panel B, middle). There were also no differences between post-GIF1 and post-GIF2 (*t* (306) = 1.301, *P* = 0.194) or post-GIF1 and post-GIF3 (*t* (304) = -1.589, *P* = 0.113). There was a significant improvement in intention from baseline to post-GIF2 phase (*t* (306) = 2.482, *P* = 0.014) and worsening between post-GIF2 and post-GIF3 phases (*t* (305) = -2.445, *P* =  0.015).

Pairwise comparisons of outcome expectancy scores revealed that, relative to baseline, ratings significantly improved in the post-GIF1 (*t* (306) = 3.006, *P* = 0.003*), post-GIF2 (*t* (307) = 3.360, *P* = 0.001*) and post-GIF3 (*t* (306) = 2.449, *P* = 0.015) phases (see **Figure 2**, panel B, right). There was no significant improvement in outcome expectancy from post-GIF1 to post-GIF2 phase (*t* (306) = 0.648, *P* = 0.517) or post-GIF2 to post-GIF3 phase (*t* (306) = -1.301, *P* = 0.194), nor was there a difference in outcome expectancy between post-GIF1 and post-GIF3 phases (*t* (305) = -0.324, *P* = 0.746).

Pairwise comparisons of self-efficacy scores revealed that, relative to baseline, self-efficacy significantly improved in post-GIF1 (*t* (307) = 2.230, *P* = 0.026) and post-GIF2 (*t* (308) = 2.812, *P* = 0.005*) phases (see **Figure 2**, panel B, left). Self-efficacy did not change from post-GIF1 to post-GIF2 phases (*t* (307) = 0.185, *P* = 0.853) and there was a significant worsening between post-GIF1 and post-GIF3 phases (*t* (306) = -2.031, *P* = 0.043), and post-GIF2 and post-GIF3 phases (*t* (307) = 2.421, *P* = 0.016). Additionally, there was no overall difference between baseline and post-GIF3 data (*t* (307) = 0.632, *P* = 0.528).

#### Mask-wearing

Pairwise comparisons of self-efficacy scores revealed a significant improvement in self-efficacy from baseline to post-GIF1 (*t* (308) = 7.775, *P* < 0.001*), post-GIF2 (*t* (308) = 8.177, *P* < 0.001*) and post-GIF3 (*t* (308) = 8.168, *P* < 0.001*) phases (see **Figure 2**, panel C, left). There was no difference in self-efficacy between post-GIF1 and post-GIF2 phases (*t* (308) = 1.878, *P* = 0.061), post-GIF1 and post-GIF3 phases (*t* (308) = 1.802, *P* = 0.073), or post-GIF2 and post-GIF3 phases (*t* (308) = -0.275, *P* = 0.783).

Pairwise comparisons of intention scores revealed the cause of the main effect of exposure was the significant improvement in intention from baseline to post-GIF1 (*t* (308) = 2.788, *P* = 0.006*), post-GIF2 (*t* (308) = 2.853, *P* = 0.005*) and post-GIF3 (*t* (308) = 4.351, *P* < 0.001*) phases (see **Figure 2**, panel C, middle). There was no difference in intention between the post-GIF1 and post-GIF2 phases (*t* (308) = 0.349, *P* = 0.727) or post-GIF2 and post-GIF3 (*t* (308) = 1.704, *P* = 0.089), but we did observe a significant difference between the post-GIF1 and post-GIF3 phases (*t* (308) = 2.070, *P* = 0.039).

Pairwise comparisons of outcome expectancy scores revealed no significant difference in scores between baseline and post-GIF1 (*t* (305) = -0.316, *P* = 0.752), post-GIF2 (*t* (305) = 0.949, *P* = 0.344), or post-GIF3 (*t* (305) = 0.780, *P* = 0.436) phases (see **Figure 2**, panel C, right). There were also no differences between post-GIF1 and post-GIF2 phases (*t* (304) = 1.266, *P* = 0.206), post-GIF2 and post-GIF3 phases (*t* (304) = 1.258, *P* = 0.209), and post-GIF2 and post-GIF3 phases (*t* (304) = -0.174, *P* = 0.862).

## DISCUSSION

Many GIFs related to the health, political and social aspects of COVID-19 have been circulating on social media as a communication device. However, despite public health agencies having used this medium to disseminate preventive messages about various health issues ranging from antibiotic resistance to heart disease and mosquito-borne diseases, GIF messaging to promote COVID-19 preventive behaviours has been surprisingly underutilized, and an evaluation of its effectiveness in shifting cognitive beliefs around these behaviours correspondingly scant. Our study sought to address these gaps in public health communication intervention design and evaluation. We found that GIFs were associated with an increase in intentions, self-efficacy, and outcome expectancy beliefs around three COVID-19 preventive behaviours – distancing, handwashing, and mask-wearing. In this section, we discuss these findings in the context of regulation surrounding these behaviours in Guatemala, reflect on study limitations, and present implications of public health communication policy and practice.

Of the three preventive behaviours, exposure to the GIFs had the most pronounced effect on distancing. This finding needs to be considered in the context of the shifts in policies surrounding distancing; the Guatemalan government published a decree called a ‘State of Calamity’ in March 2020 [28] which legally enforced preventive behaviours, including distancing of 1.5 m in public places [29]. This decree was withdrawn in September 2020 [30] to allow businesses to reopen and it remained withdrawn during the study period (February-March 2021) when the public were ‘urged’ to practice distancing [31]). Our finding can possibly be explained by the low baseline levels of self-efficacy around distancing, given the challenges of practising it in the face of overcrowding and the economic hardships it imposed [28]. Consequently, it appears that study respondents were reconciling the need for practising distancing to prevent COVID-19 transmission with its potential downsides. Similar barriers to practising distancing including social responsibilities, lack of trust in the government, and stress due to isolation have been observed elsewhere [32]. Ensuring public adherence to distancing is a complex socio-behavioural intervention that requires strategic messaging and structural reconfiguration of social spaces [33]. This complexity is reflected in low self-efficacy among the public and opens the need for communication interventions that visually demonstrate behaviours, providing vicarious learning opportunities as set out in SCT [14].

At the time of data collection, the Guatemalan public were required to wear face masks in public places [31]. This requirement, coupled with baseline data indicating that 91% of participants reported consistent mask-wearing in the week preceding the study and 81% strongly felt that not wearing a mask correctly would increase transmission risk, suggest that respondents might have habituated to the behaviour. Consequently, we observed relatively higher levels of self-efficacy and a potential ceiling effect for outcome expectancies with minimal room for increase through further communication interventions.

We observed similar trends in relation to handwashing, with a potential ceiling effect for self-efficacy. Handwashing is more commonplace than mask-wearing and respondents are likely to have had frequent mastery experiences. In situations where baseline beliefs are high, messages like those used in this study may serve to reinforce beliefs and strengthen intentions.

While the combinations in which GIFs were presented bore minimal impact, what commands our attention is the dosage of GIF exposure. Across the three behaviours, where improvements were observed, they were most pronounced from baseline to early exposures, after which they plateaued. In some cases, such as those of self-efficacy for handwashing and outcome expectancy for handwashing and distancing, we observed slightly (statistically insignificant) counter-productive outcomes suggesting that calibrating the dosage of communication might be a critical consideration for health communication. User fatigue, which can explain discontinued social media use [34], can be considered as important to this end.

These findings must be considered against three main limitations of our study. One, a cross-sectional study design offers useful insights at one point in time, but beliefs around such behaviours may shift over time as part of their ‘natural history’ or in response to the state of the pandemic and current government guidance. Two, we collected data on immediate effects of GIF exposure and therefore cannot comment on the durability or resilience of effects. Three, we report effects in terms of associations between GIF exposure and theoretical and evidence-based determinants of preventive behaviours; therefore, more research is needed to identify associations with adherence to the behaviours.

## CONCLUSION

Our study offers three new insights for public health communication researchers and practitioners. First, languageless GIFs offer public health agencies a valuable and potentially cost-effective multimedia tool to widely disseminate messages via social media in multilingual countries during health crises such as COVID-19; their contribution to global “vaccines-plus” action [35] to communicate vaccination and preventive public health measures for COVID-19 should be recognised and welcomed. Second, the effectiveness of GIF interventions may be affected by prevailing levels of health beliefs, which suggests that understanding public sentiment prior to their design and dissemination might be important. Third, while the seeming ease of scaling GIF interventions might tempt an “all-out” approach in terms of quantity, more might not necessarily be better. Specifically, it is important to strategically calibrate the dosage in which GIFs are delivered, bearing in mind that their ability to play in a ‘looping repetition’ [19] can reinforce messages, using approaches from simple awareness to satire and humour. Finally, we recommend that health and risk communication researchers and practitioners adapt our evaluation – preferably through more scaled-up, representative designs – in different countries, to examine how local restrictions and cultural variables might mediate the associations between GIF exposure and behavioural intentions and beliefs.
